# Therapeutic Potential of Intra-Articular Delivery of Allogeneic Dedifferentiated Fat Cells in a Canine Monoiodoacetate-Induced Knee Osteoarthritis Model

**DOI:** 10.3390/cells15171585

**Published:** 2026-08-31

**Authors:** Rei Nakano, Azusa Seki, Hiroshi Sugiya

**Affiliations:** 1 LUMIRISE Inc., 2-6-2 Shinkawa, Chuo, Tokyo 104-0033, Japan; sugiya.hiroshi@nihon-u.ac.jp; 2 Japan Animal Specialty Medical Institute, 1-8-37, Nakagawa, Tsuzuki, Yokohama 224-0001, Kanagawa, Japan; 3 Laboratory for Mucosal Immunity, RIKEN Center for Integrative Medical Sciences, 1-7-22 Suehiro-cho, Tsurumi, Yokohama 230-0045, Kanagawa, Japan; 4 Fukushima Medical Device Development Support Centre, 27-8 Mansuida, Tomitamachi, Koriyama 963-8041, Fukushima, Japan

**Keywords:** dedifferentiated fat cells, canine model, osteoarthritis, large animal model, allogenic transplantation

## Abstract

**Highlights:**

**What are the main findings?**

- DFAT Cell Efficacy: Intra-articular injection of allogeneic dedifferentiated fat cells reduced cartilage erosion and osteophyte formation (lower OARSI scores) in a canine MIA-induced osteoarthritis model.
- Matrix Preservation: Histological evaluation (toluidine blue staining) demonstrated that DFAT cell therapy markedly preserved extracellular matrix components and proteoglycan retention compared to the vehicle group.

**What are the implications of the main findings?**

- Translational Relevance: Utilizing a canine model—which closely mirrors human joint biomechanics, cartilage thickness, and spontaneous disease progression—bridges the gap between preclinical rodent studies and human clinical applications.
- Therapeutic Potential: These results highlight allogeneic DFAT cells as a promising stem cell-based regenerative therapy for treating chronic, naturally occurring osteoarthritis in both veterinary and human medicine.

**Abstract:**

Osteoarthritis (OA) is the leading cause of disability worldwide. Although rodent models are widely used in OA research, the disease is often artificially accelerated in these systems, and joint biomechanics differ substantially from those of humans. In contrast, canine models offer greater translational relevance due to similarities in joint size, cartilage thickness, and physiological mechanical loading of the knee, making them suitable surrogates for evaluating regenerative therapies. In this study, we assessed the therapeutic efficacy of allogeneic dedifferentiated fat (DFAT) cells, which exhibit greater cellular homogeneity and enhanced osteochondrogenic potential compared with conventional mesenchymal stem cells (MSCs), in a canine monoiodoacetate (MIA)-induced OA model. OA was induced by intra-articular (i.a.) injection of MIA into the knee joints of beagles. Animals were allocated to three groups: an implant group receiving allogeneic DFAT cells via i.a. injection, a vehicle group, and a sham group. Therapeutic outcomes were evaluated through macroscopic scoring of cartilage surfaces and histological assessment using toluidine blue staining to examine proteoglycan depletion and structural integrity. The implant group demonstrated significantly lower Osteoarthritis Research Society International (OARSI) scores for cartilage erosion and osteophyte formation compared with the vehicle group, indicating preservation of joint surface integrity. Toluidine blue staining further showed that DFAT cell implantation significantly preserved extracellular matrix components, with greater proteoglycan retention relative to vehicle-treated controls. A key rationale for employing a canine model was the high prevalence of spontaneous OA in dogs, which closely resembles human disease in clinical presentation, radiographic findings, and molecular pathways. Unlike rodent models, dogs naturally develop OA with progressive degenerative features that mirror human conditions. By utilizing a large animal model, this study bridges the gap between preclinical in vitro findings and potential human clinical application. Collectively, these results provide strong evidence supporting the therapeutic potential of allogeneic DFAT cells and suggest that DFAT-based therapy may represent a promising treatment strategy for chronic, naturally occurring OA in both veterinary and human patients.

## 1. Introduction

Osteoarthritis (OA) is a chronic, degenerative joint disease characterized by the progressive destruction of articular cartilage, resulting in pain, swelling, stiffness, reduced range of motion, and lameness. The pathogenesis of OA is multifactorial and is influenced by both physiological and mechanical factors, including age and obesity.

Current therapeutic approaches—such as controlled exercise, physical rehabilitation, and non-steroidal anti-inflammatory drugs—are primarily aimed at symptomatic management. Although these strategies are essential for maintaining patient quality of life, their effects are temporary and do not halt or reverse the structural progression of the disease, including cartilage degradation and joint dysfunction. Consequently, regenerative strategies, particularly cell-based therapies, have gained attention as potential disease-modifying interventions capable of restoring joint structure and function. Because aging and obesity negatively affect the quality and regenerative capacity of autologous cell sources, allogeneic cell-based therapies offer a more consistent and potentially superior alternative that is not compromised by the patient’s systemic condition [1].

From a translational perspective, large animal models—especially canine models—are critical for bridging the gap between in vitro research and human clinical application [2]. Dogs provide substantial advantages over rodent models due to their closer resemblance to humans in joint anatomy, cartilage thickness, and biomechanical loading of weight-bearing joints [2]. Furthermore, the high prevalence of naturally occurring OA in dogs, which recapitulates the clinical presentation, radiographic features, and pathological progression observed in humans, further supports their relevance as translational models for evaluating regenerative therapies [3].

Mesenchymal stem cells (MSCs) have been extensively investigated for cartilage repair; however, bone marrow- and adipose-derived MSCs are often limited by cellular heterogeneity and restricted proliferative capacity. To address these limitations, dedifferentiated fat (DFAT) cells have been introduced as a novel progenitor cell source. DFAT cells are derived from mature adipocytes through a ceiling culture technique, yielding a highly homogeneous population with robust self-renewal capacity and strong osteochondrogenic potential [4,5]. Previous studies have demonstrated that DFAT cells exhibit improved phenotypic stability and scalability in manufacturing compared with conventional stromal vascular fraction-derived MSCs—attributes that are particularly important for standardized pharmaceutical development [6].

In the present study, we investigated the therapeutic efficacy of intra-articular (i.a.) administration of allogeneic DFAT cells in a canine monoiodoacetate (MIA)-induced OA model. We demonstrate that allogeneic DFAT transplantation effectively attenuates cartilage degradation and promotes preservation of the extracellular matrix within a large-animal joint environment. These findings provide a strong translational basis for the clinical development of allogeneic DFAT cell therapy for OA.

## 2. Materials and Methods

### 2.1. Materials

Collagenase II was purchased from Sigma-Aldrich Inc. (St. Louis, MO, USA). STEM-CELLBANKER GMP grade was obtained from TaKaRa Bio, Inc. (Kusatsu, Shiga, Japan). Trypsin-EDTA and α-minimum essential medium (α-MEM) were purchased from Wako Pure Chemical Industries Ltd. (Osaka, Japan). Fetal bovine serum (FBS) was obtained from Biowest (Nuaillé, France). A controlled-rate freezing vessel (BICELL) was purchased from Nihon Freezer Co., Ltd. (Tokyo, Japan).

### 2.2. Primary DFAT Cell Cultures

Three healthy male beagle dogs (1 year old; KITAYAMA LABES Co., Ltd., Nagano, Japan) were used for DFAT cell isolation. DFAT cells were isolated using the ceiling culture method with minor modifications, as previously described [4,5,6,7,8,9,10]. Briefly, canine celiac adipose tissue was minced in α-MEM containing 0.1% collagenase II and 2% FBS. Samples were incubated at 37 °C for 30 min with gentle agitation and centrifuged at 500× *g* for 3 min. The floating layer containing mature adipocytes was collected and washed three times. Cells were transferred to 25-cm^2^ culture flasks and maintained under ceiling culture conditions in α-MEM supplemented with 10% FBS at 37 °C in 5% CO_2_. After three days, cultures were maintained under standard conditions prior to cryopreservation. Media was changed twice weekly. Cells were harvested at approximately 90% confluence using 0.25% trypsin-EDTA and reseeded at 1 × 10^6^ cells per 75-cm^2^ flask. Fourth-passage cells were used for all subsequent experiments.

### 2.3. Cell Cryopreservation

Cells were harvested at 90–95% confluence using 0.25% trypsin-EDTA. Collected cells were suspended in STEM-CELLBANKER^®^ GMP grade at a density of 5 × 10^6^ cells per 500 μL, and 500 μL aliquots were dispensed into sterile serum tubes as previously described [11,12,13,14,15,16,17,18,19,20,21,22,23]. Tubes were placed in a BICELL freezing vessel and cryopreserved at −80 °C. Prior to transplantation, cryopreserved sample tubes were rapidly thawed in a 37 °C water bath. The cell suspension was diluted with 0.9% NaCl and centrifuged at 300× *g* for 3 min. After removal of the supernatant, the pellet was resuspended in sterile 0.9% NaCl saline for injection.

### 2.4. Ethics Statement

All animal procedures were approved by the Institutional Animal Care and Use Committee of HAMRI Co., Ltd. (Koga, Japan) (Approval No. 16-H051 for DFAT cell isolation [Approval Date: 1 September 2016], Approval No. 23-H105 for induction of knee osteoarthritis dog model [Approval Date: 29 August 2023]).

### 2.5. Induction of Knee Osteoarthritis

**Figure 1:**
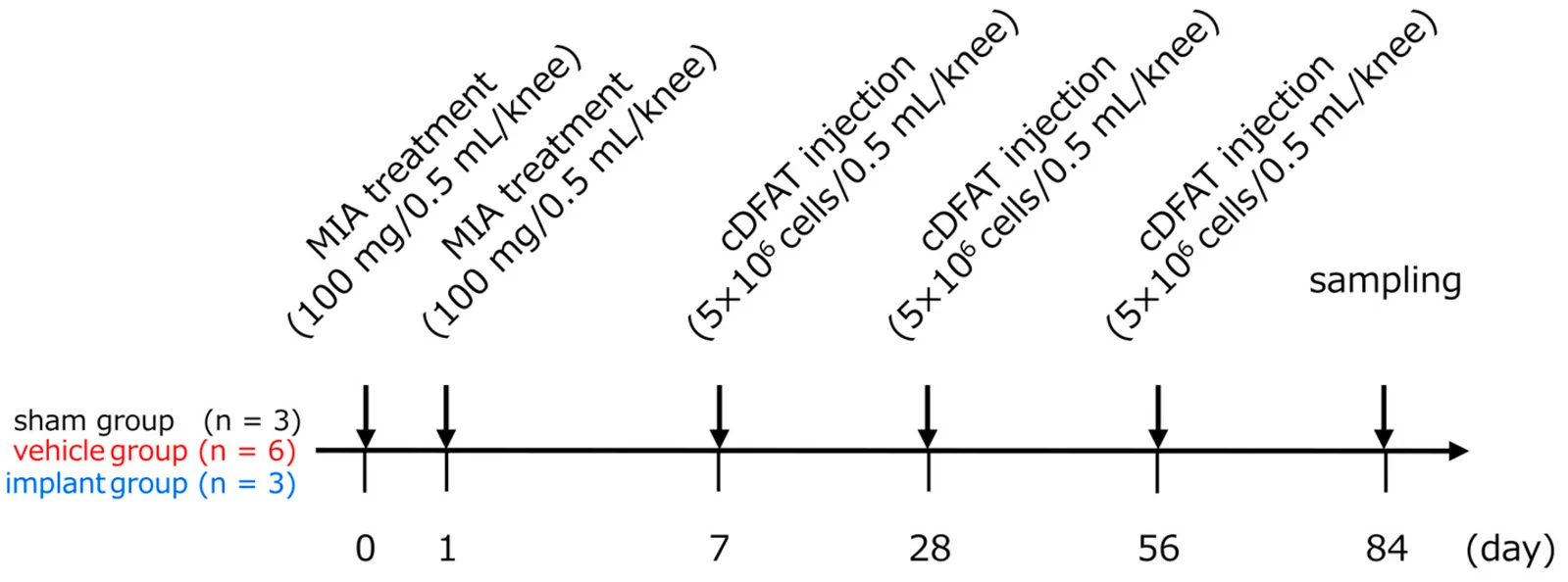
Study design and clinical evaluation timeline. Schematic representation of the experimental protocol for osteoarthritis (OA) induction and intra-articular (i.a.) administration of canine dedifferentiated fat (DFAT) cells. OA was induced in beagle dogs by i.a. injection of sodium monoiodoacetate (MIA; 100 mg/0.5 mL per knee joint) on days 0 and 1. The implant group received i.a. injections of canine DFAT cells (cDFAT; 5 × 10^6^ cells/0.5 mL per knee) at 7, 28, and 56 days following MIA induction. Joint tissues were harvested for macroscopic and histological analyses on day 84.

Twelve healthy male beagles (1 year old; Kitayama Labes Co., Ltd., Nagano, Japan) were used. Animals were randomly assigned to three groups: sham (n = 3), vehicle (n = 6), and implant (n = 3). Knee OA was induced via repeated i.a. injections of MIA (Nacalai Tesque Inc., Kyoto, Japan) into both sides of the knee joint as previously described [24,25,26] (Figure 1). Prior to injection, knees were shaved and disinfected with povidone-iodine. In the vehicle and implant groups, 100 mg MIA dissolved in sterile 0.9% NaCl saline (0.5 mL) was injected into the joint space via the infrapatellar ligament using a 27-gauge needle. The sham group received 0.5 mL sterile saline via the same approach. Injections were performed once daily for two consecutive days, resulting in a total MIA dose of 200 mg per joint. Knee OA validation and quality control were confirmed prior to the primary experiments using a separate satellite cohort. In these pilot studies, dogs subjected to Knee OA exhibited a significant increase in clinical score. This quality control checkpoint confirmed the functional efficacy and uniformity of our knee OA model before cell delivery.

### 2.6. DFAT Cell Transplantation

Following OA induction, the implant group received i.a. injection of allogeneic DFAT cells (5 × 10^6^ cells in 500 μL sterile 0.9% NaCl saline per joint) at 7, 28, and 56 days post-MIA administration. Allogeneic canine DFAT from each donor was administered separately to individual recipient animals without pooling, thereby maintaining donor identity across the treatment cohort. Cells were delivered using a 27-gauge needle via the infrapatellar ligament. The vehicle group received equivalent volumes of 0.9% NaCl sterile saline at identical time points.

### 2.7. Efforts to Alleviate Suffering

Dogs were housed individually in cages (height 75 cm; width 80 cm; length 75 cm). Animals received experimental food (DS-A, 250 g/head/day; Oriental Yeast Co., Ltd., Tokyo, Japan) once daily. To minimize animal suffering and distress, all dogs were provided with environmental enrichment, including social interaction and daily exercise. Exercise was provided using toys inside the facility (daily) and outside the breeding facility (monthly). Facility conditions were monitored daily. To prevent infection, animals were housed separately. All efforts were made to minimize discomfort. All research staff were certified in laboratory animal science and had specific training in the handling and clinical assessment of Beagle dogs, as mandated by the Institutional Animal Care and Use Committee of HAMRI Co., Ltd.

### 2.8. Methods of Analgesia

To ensure animal welfare and minimize postoperative distress, all Beagle dogs received standardized pharmacological support. For pain management, buprenorphine hydrochloride (Lepetan 0.2 mg injection; Otsuka Pharmaceutical Co., Ltd., Tokyo, Japan) was administered intramuscularly at a dose of 20 µg/0.1 mL/kg once daily for seven consecutive days following i.a. administration. To prevent secondary infections, enrofloxacin (Baytril 2.5% injection; Elanco Japan K.K., Tokyo, Japan) was administered intramuscularly at 10 mg/0.4 mL/kg for the same seven-day period. Throughout the recovery phase, the animals were closely monitored for any adverse reactions to the medications or signs of breakthrough pain.

### 2.9. Humane Endpoints and Monitoring

Animals were monitored daily by trained staff for clinical signs of pain or distress, such as decreased appetite, lethargy, abnormal gait, or vocalization. The humane endpoint was predefined as meeting any of the following criteria: (1) body weight loss exceeding 15% compared to the baseline, (2) inability to maintain an upright posture or impaired mobility, (3) severe respiratory distress (e.g., labored breathing or cyanosis), or (4) persistent anorexia for more than 24 h. If any animal met these criteria, it was immediately euthanized to prevent further suffering. In this study, no dogs reached the humane endpoint, and twelve dogs were euthanized at the planned experimental endpoint. No animals died before reaching the humane endpoint.

### 2.10. Methods of Sacrifice

Upon completion of the experimental period, all animals were humanely euthanized using an intravenous overdose of thiopental sodium (50 mg/2 mL/kg; Isozol 0.5 g, Nichi-Iko Pharmaceutical Co., Ltd., Toyama, Japan). To ensure compliance with the AVMA Guidelines for the Euthanasia of Animals, the irreversible cessation of both heartbeat and respiration was confirmed in all subjects.

### 2.11. Clinical Evaluation

Clinical status was longitudinally monitored to evaluate safety and functional outcomes. Body weight (kg) was recorded on days 0, 7, 28, 56, and 84 after MIA induction. Joint swelling was semi-quantitatively scored (0–3 scale: 0 none; 1 mild; 2 moderate; 3 severe) for up to 60 days, by which time physical swelling scores had stabilized and returned to baseline levels across all experimental groups. Lameness (days 7 and 30 post-MIA) and gait abnormalities (days 7 and 60 post-MIA) were also scored on a 0–3 scale. All evaluations were conducted by two independent observers blinded to group allocation. Data exclusion was strictly governed by predefined criteria set prior to the experiments to eliminate confounding technical and behavioral artifacts. Regarding technical exclusions, behavioral anomalies or non-engagement, no dogs were excluded in this study.

### 2.12. Macroscopic Assessment

At the study endpoint (day 84), knee joints were harvested. Articular surfaces of the femur (trochlea, medial, and lateral condyles) and tibia (medial and lateral plateaus) were examined macroscopically. Cartilage lesions were graded according to the OARSI macroscopic scoring system for canine models [3], assessing surface integrity, erosion extent, and osteophyte formation. Total macroscopic scores were calculated as the sum of all evaluated joint regions to provide a comprehensive assessment of joint degeneration [3].

### 2.13. Histological Processing and Quantification

Samples were fixed in 10% neutral-buffered formalin, decalcified, and paraffin-embedded. Sagittal sections (5 μm) were prepared and stained with hematoxylin and eosin (H&E) for structural evaluation. Toluidine blue staining was performed to assess proteoglycan content. For quantitative analysis, stained areas were measured using a standardized digital image-processing pipeline implemented in Python with OpenCV (version 4.x). High-resolution images were converted from RGB to hue-saturation-value (HSV) color space to enable more robust color segmentation independent of illumination variation. Toluidine blue-positive regions were segmented using predefined thresholds (hue 110–170; saturation 30–255; and value 20–230). Morphological opening (5 × 5 pixel kernel) was applied to remove background noise and artifacts. The total stained area was quantified by summing pixels within the binary mask, representing proteoglycan-rich extracellular matrix. Individual sections were analyzed separately. Pixel counts were used for statistical comparison of chondroprotective effects.

### 2.14. Synovial Fluid Prostagandin E_2_ (PGE_2_) Quantification

Synovial fluid samples collected at the study endpoint were stored at −80 °C until analysis. The concentration of PGE_2_ was determined using a commercially available canine-validated PGE_2_ enzyme-linked immunosorbent assay (ELISA) kit (Cayman Chemical Co., Ann Arbor, MI, USA) according to the manufacturer’s instructions.

### 2.15. Randomization, Allocation Concealment, and Blinding

Dogs were randomized into experimental groups. To maintain allocation concealment, an independent researcher prepared the transplantation materials in identical, coded vials. Accordingly, a single researcher performed all surgical procedures to properly and consistently assess the biological variability between the models while remaining completely blind to the specific treatment groups. Furthermore, all downstream analyses were performed by independent investigators who were blinded to the experimental groups.

### 2.16. Quantification and Statistical Analysis

**Figure 2:**
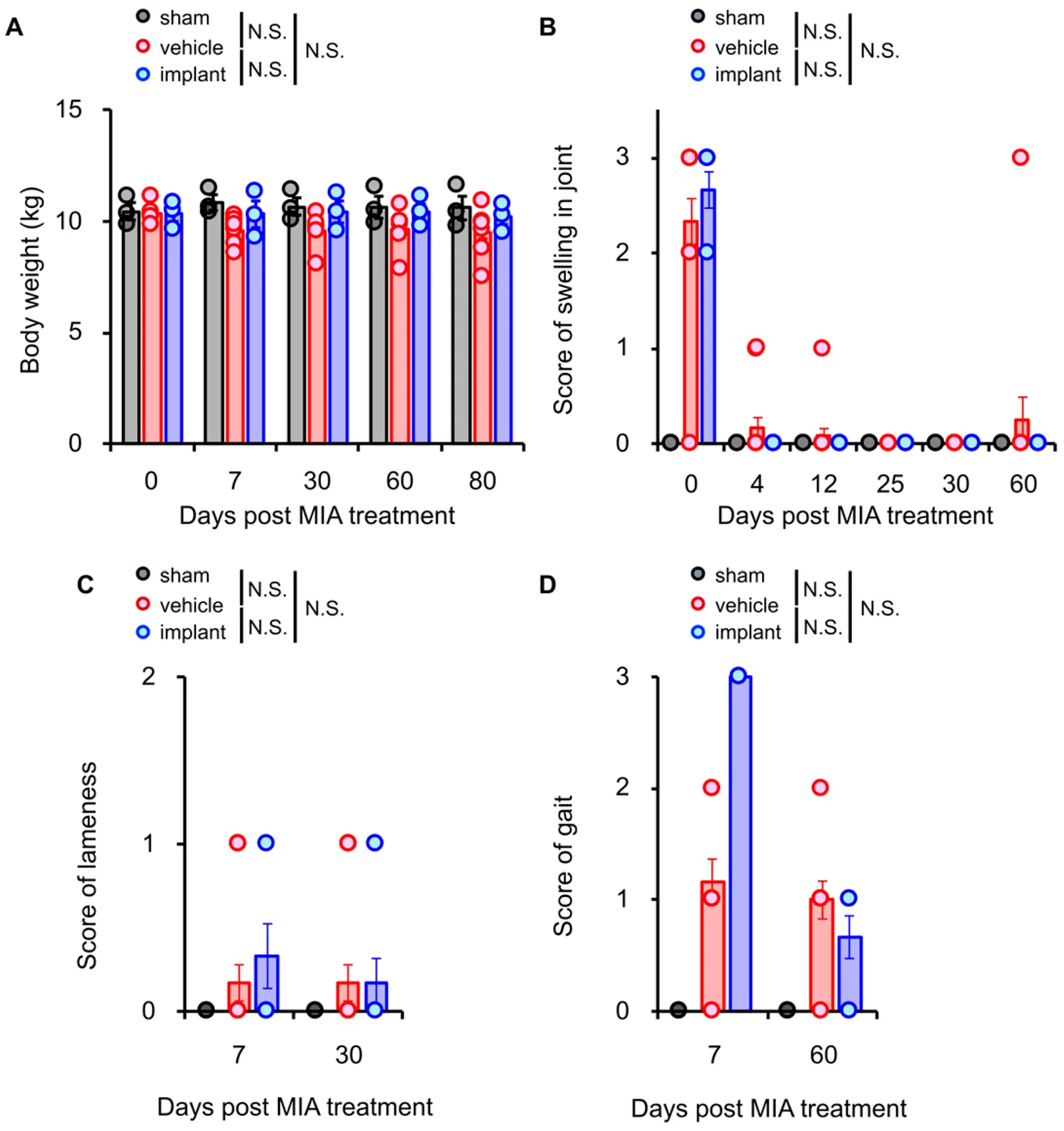
Systematic and local safety of intra-articular allogeneic DFAT cell transplantation. (**A**) No significant differences in body weight were observed among the sham, vehicle, and implant groups over the 84-day period, indicating absence of systemic toxicity. (**B**) Acute joint swelling was observed following MIA administration on day 0 in both the vehicle and implant groups. Swelling subsided by day 7 in the implant group and remained at baseline thereafter. Although the implant group showed consistently lower swelling scores than the vehicle group at later time points, the differences were not statistically significant. The vehicle group exhibited minor late-stage fluctuations around day 60. (**C**) Mild lameness was detected following OA induction. By day 30, the implant group demonstrated a downward trend in lameness scores relative to the vehicle group; however, the differences did not reach statistical significance. (**D**) Gait scores increased transiently in both the vehicle and implant groups following MIA induction and gradually declined over time. The implant group showed a clearer return toward baseline by day 60, although no significant intergroup differences were observed. Data are expressed as mean ± SEM. N.S., not significant, as determined by Kruskal–Wallis rank sum test followed by Mann–Whitney U test with the Holm–Bonferroni adjustment.

**Figure 3:**
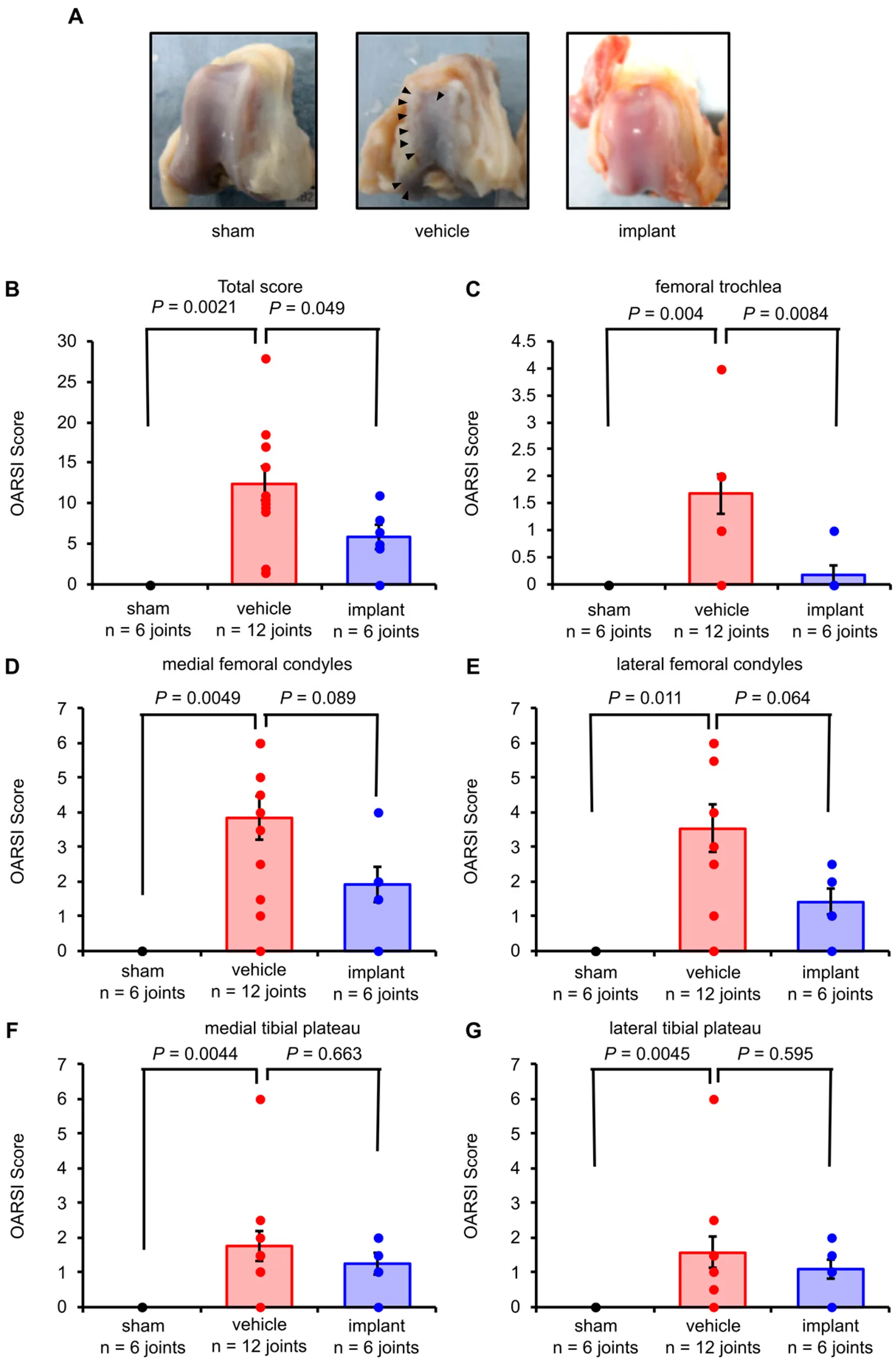
Macroscopic scoring of cartilage degeneration. (**A**) Representative macroscopic images of the femoral articular surfaces. The sham group shows smooth, intact cartilage. The vehicle group exhibits characteristic osteoarthritic changes, including surface fibrillation, erosion, and cartilage loss (black arrowheads). The implant (DFAT-treated) group demonstrates reduced cartilage attrition and preservation of articular surface integrity compared with the vehicle group. (**B**) Total OARSI score. Regional sub-scores include (**C**) OARSI score of femoral trochlea, (**D**) OARSI score of medial femoral condyles, (**E**) OARSI score of lateral femoral condyles, (**F**) OARSI score of medial tibial plateau, and (**G**) OARSI score of lateral tibial plateau. The implant group showed a significant reduction in total OARSI score compared with the vehicle group. In the femoral trochlea (**C**), DFAT treatment significantly attenuated cartilage degeneration. Although lower mean scores were observed in the medial and lateral femoral condyles (**D**,**E**) in the implant group, these differences were not statistically significant. No significant differences were detected between the vehicle and implant groups in the tibial plateau regions (**F**,**G**). Data are presented as mean ± SEM with individual data points shown (sham, n = 6 joints from N = 3 animals; vehicle, n = 12 joints from N = 6 animals; implant, n = 6 joints from N = 3 animals). Statistical significance was determined by a Kruskal–Wallis rank sum test followed by a Mann–Whitney U test with the Holm–Bonferroni adjustment.

**Figure 4:**
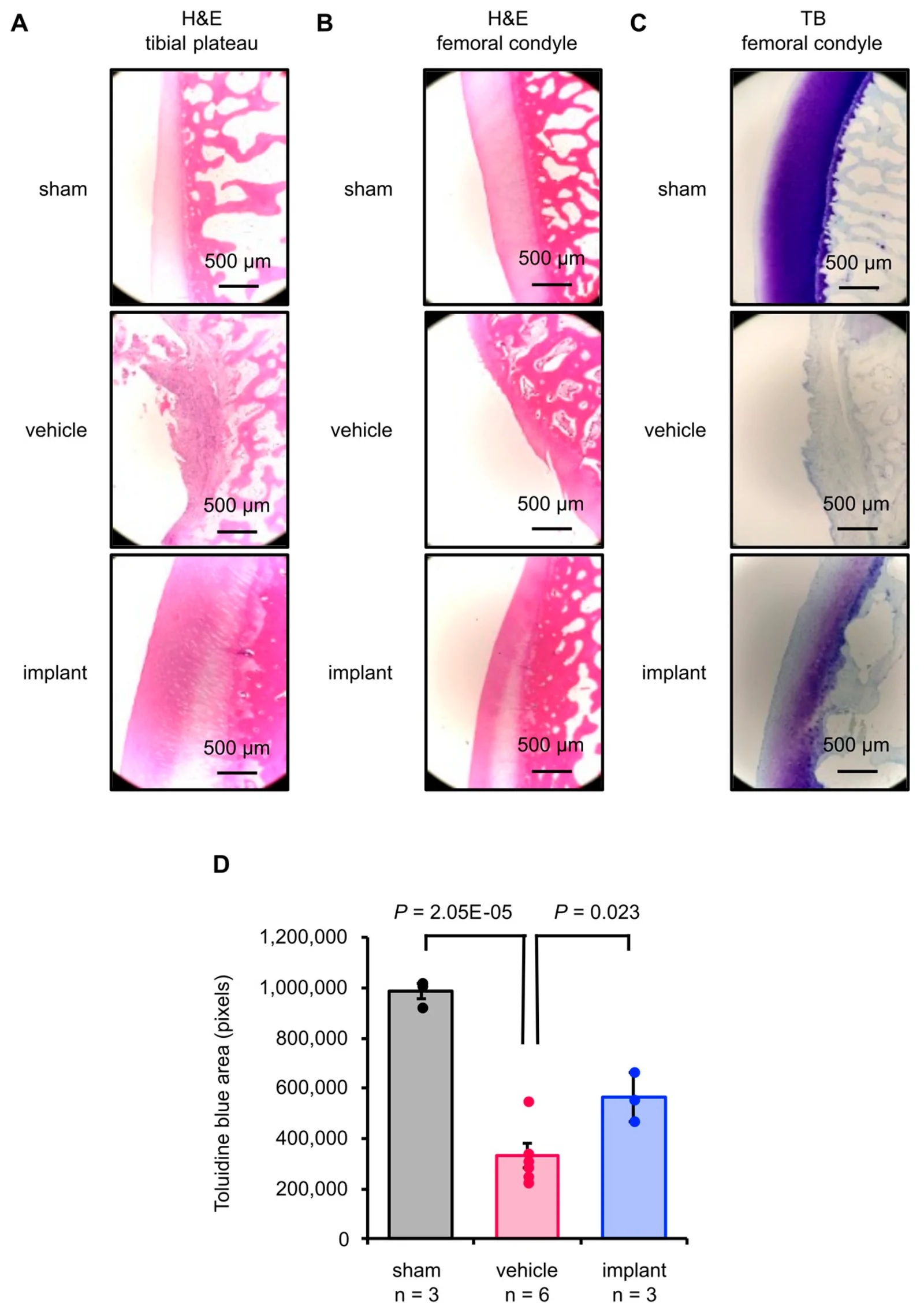
Histological evaluation of cartilage integrity and proteoglycan content. (**A**,**B**) Representative histological sections of the tibial plateau (**A**) and femoral condyle (**B**). In hematoxylin and eosin staining (H&E), the sham group exhibits a smooth articular surface and organized chondrocyte architecture. The vehicle group demonstrates structural deterioration, including surface irregularities and cartilage thinning. The implant (DFAT-treated) group shows improved structural preservation relative to the vehicle group. (**C**) In toluidine blue staining (TB), intense metachromasia in the sham group reflects abundant sulfated glycosaminoglycans and intact extracellular matrix (ECM). The vehicle group displays marked loss of metachromasia, indicating severe matrix depletion. The implant group demonstrates partial restoration of staining intensity, consistent with attenuation of ECM degradation. Scale bars = 500 μm. (**D**) Quantitative analysis of toluidine blue-positive area. The vehicle group showed a significant reduction in proteoglycan-positive area compared with the sham group, confirming successful OA induction. The implant group exhibited a significant increase in stained area relative to the vehicle group, indicating preservation of cartilage ECM following DFAT transplantation. Data are expressed as mean ± SEM with individual data points shown (sham, n = 3; vehicle, n = 6; implant, n = 3). Data passed the normality test (Kolmogorov–Smirnov test: D = 0.178, *p* = 0.780; Shapiro–Wilk test: W = 0.877, *p* = 0.080). Bartlett’s test indicated no significant difference in variance among the three groups (χ^2^ = 1.144, *p* = 0.565). Statistical significance was determined by one-way ANOVA followed by Tukey’s post hoc test.

**Figure 5:**
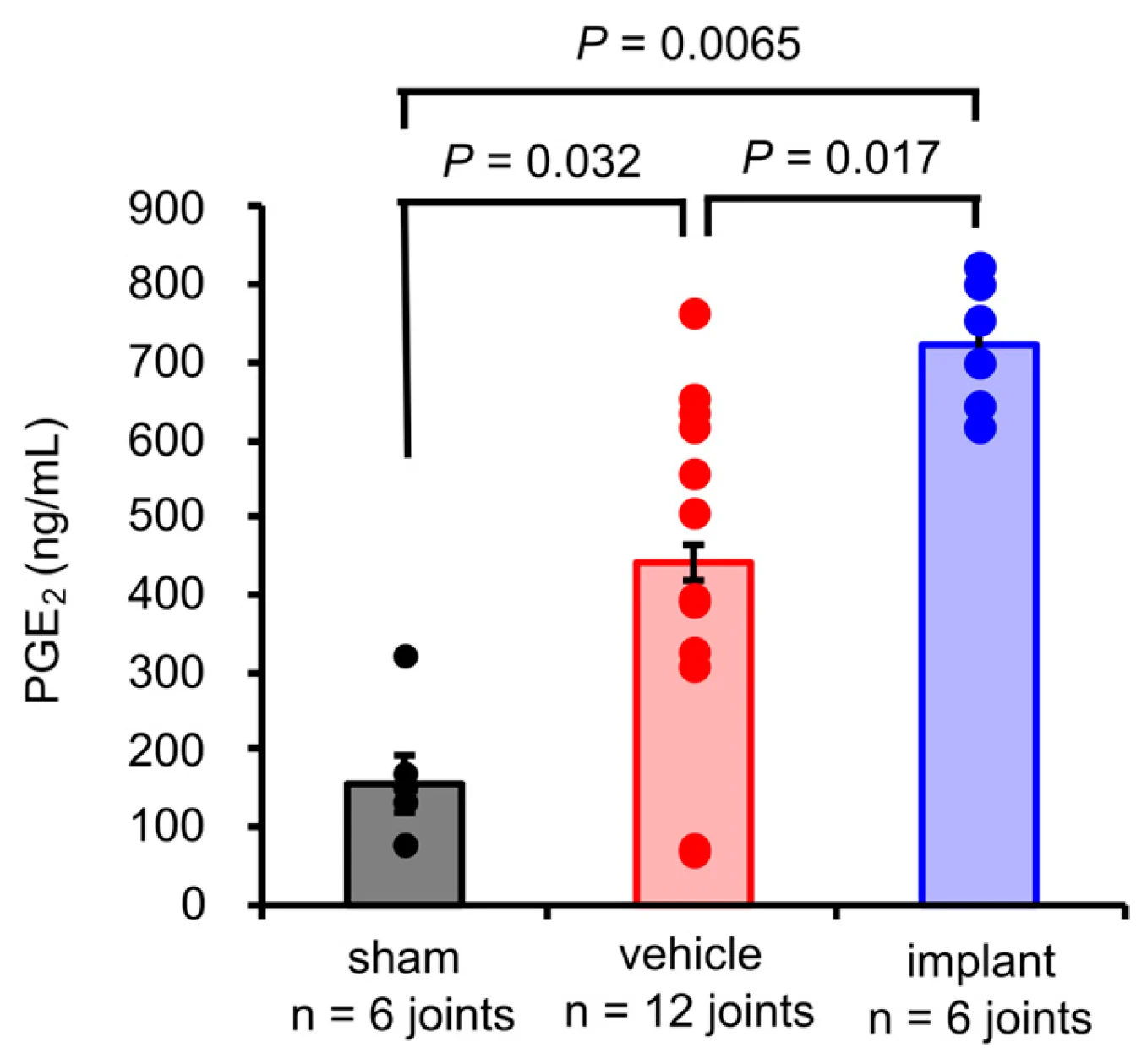
Quantification of prostaglandin E_2_ (PGE_2_) in synovial fluid. Concentrations of PGE_2_ in synovial fluid collected at Day 84 post-MIA administration were determined by ELISA for the sham, vehicle, and implant groups. Data are presented as mean ± SEM with individual data points (sham, n = 6 joints from N = 3 animals; vehicle, n = 12 joints from N = 6 animals; implant, n = 6 joints from N = 3 animals). Statistical significance was determined by the Kruskal–Wallis rank sum test followed by the Mann–Whitney U test with the Holm–Bonferroni adjustment.

Statistical analyses were performed using EZR software (Ver. 1.68) [27]. Data are presented as mean ± standard error (SEM). Statistical significance was determined by the Kruskal–Wallis rank sum test followed by the Mann–Whitney U test, and the Holm–Bonferroni adjustment was applied to OARSI score and PGE_2_ (Figure 2A–D, Figure 3B–G and Figure 5). Statistical significance was determined by one-way ANOVA followed by Tukey’s post hoc test as shown in Figure 4D. A *p*-value < 0.05 was considered statistically significant. A post hoc power analysis was conducted using G*Power software (version 3.1.9.7) for the overall group comparison (one-way ANOVA framework) of the primary endpoint (total OARSI histopathology score). Based on the observed effect size (Cohen’s f = 0.988), a significance level of α = 0.05, and a total sample size of n = 24 joints (n = 6 joints for sham group, n = 12 for vehicle group, and n = 6 for implant group), the achieved statistical power (1 − β) was 0.986 (98.6%).

## 3. Results

### 3.1. Systemic and Local Safety of Intra-Articular Allogeneic DFAT Cell Transplantation in MIA-Induced OA

To evaluate the systemic and local safety of i.a. allogeneic DFAT cell transplantation, clinical parameters including body weight and joint condition were monitored throughout the study period. No significant differences in body weight were observed among the sham, vehicle, and implant groups over the 84-day experimental period (Figure 2A). All animals maintained stable body weights, indicating that i.a. administration of MIA followed by DFAT cell transplantation did not induce systemic toxicity or adverse metabolic effects. As shown in Figure 2B, i.a. injection of MIA induced acute joint swelling in both the vehicle and implant groups. However, swelling subsided within 7 days post-injection. In the implant group, swelling scores remained at baseline thereafter, whereas the vehicle group exhibited a slight, non-significant recurrence of mild swelling by day 60 (Figure 2B). No sustained inflammatory response attributable to the allogeneic DFAT cells was observed (Figure 2B). Mild lameness was detected in both the vehicle and implant groups on day 7, likely reflecting acute MIA-induced joint irritation (Figure 2C). Although lameness scores decreased by day 30 in the implant group, no statistically significant intergroup differences were observed (Figure 2C). Gait scores remained consistently low (near zero) across all groups throughout the observation period, indicating that neither OA induction nor DFAT transplantation resulted in persistent functional impairment (Figure 2D). Therefore, subsequent evaluations through Day 84 were focused on structural histopathological outcomes. At the study endpoint (Day 84), comprehensive hematological analyses were performed (Table 1). Complete blood count parameters, including white blood cell (WBC), red blood cell (RBC), and hemoglobin (HGB) levels, remained within normal reference ranges in all groups. No significant differences were detected in leukocyte subsets, including lymphocytes (LY), monocytes (MO), or granulocytes (GR). Although a statistically significant difference in eosinophil (EO) percentage was observed between the sham and implant groups (*p* = 0.043), values remained within clinically acceptable limits. Macroscopic and histopathological evaluations of major organs (heart, lung, liver, spleen, and kidney) revealed no treatment-related abnormalities (Table 2). Most tissues were classified as “nothing abnormal detected” (NAD) in all groups. Isolated findings—including mild plasmacytic cholangitis in the sham group, bronchopneumonia/hepatitis in the vehicle group, and a single case of nephrocalcinosis in the implant group—were considered incidental and unrelated to DFAT administration. Collectively, these findings demonstrate that i.a. delivery of allogeneic DFAT cells was well tolerated, with no evidence of systemic toxicity, acute rejection, persistent inflammation, or functional deterioration, supporting its safety profile for translational application.

### 3.2. Macroscopic Evaluation of Articular Cartilage

At the study endpoint, macroscopic assessment of the knee joints was performed to evaluate the chondroprotective effects of DFAT cells (Figure 3A). In the vehicle group, the articular surfaces exhibited pronounced degenerative changes, including cartilage erosion and osteophyte formation, particularly in the femoral trochlea and condyles (Figure 3A, black arrowheads). In contrast, the implant (DFAT-treated) group displayed visibly smoother articular surfaces with markedly reduced erosive lesions, closely resembling the sham group. The total macroscopic OARSI score was significantly higher in the vehicle group than that in the sham group (Figure 3B, *p* = 0.0021). Notably, DFAT treatment significantly reduced the total OARSI score compared with the vehicle group (Figure 3B, *p* = 0.049), indicating an overall protective effect against MIA-induced joint degradation. Regional analysis revealed that the most pronounced improvement occurred in the femoral trochlea, where the implant group demonstrated significantly lower OARSI scores than the vehicle group (Figure 3C, *p* = 0.0084). In the medial (Figure 3D) and lateral (Figure 3E) femoral condyles, the implant group showed lower mean scores than the vehicle group; however, these differences did not reach statistical significance (*p* = 0.089 and *p* = 0.064, respectively). The vehicle group exhibited significantly greater degeneration compared with the sham group in both regions (*p* = 0.0049 and *p* = 0.011, respectively). Similarly, significant cartilage damage was observed in the medial (Figure 3F) and lateral (Figure 3G) tibial plateaus of the vehicle group compared with the sham group (*p* < 0.005 for both). Although the implant group demonstrated slightly lower mean scores in these regions, differences between the vehicle and implant groups were not statistically significant (*p* = 0.663 and *p* = 0.595, respectively). Collectively, these macroscopic findings demonstrate that i.a. administration of allogeneic DFAT cells significantly attenuates cartilage erosion and joint degeneration, with the most substantial therapeutic effect observed in the femoral trochlea.

### 3.3. Histopathological Evaluation of Cartilage Regeneration

To further assess the chondroprotective effects of DFAT cells at the microscopic level, histological analyses were performed using H&E (Figure 4A,B) and toluidine blue staining (Figure 4C). In the vehicle group, H&E staining revealed advanced osteoarthritic changes, including vertical fissures, pronounced surface fibrillation, and substantial thinning of the cartilage layer. In contrast, the implant group preserved a relatively smooth articular surface and maintained cartilage architecture comparable to that of the sham group (Figure 4A,B). Toluidine blue staining, which reflects sulfated glycosaminoglycan (GAG) content, demonstrated near-complete loss of metachromasia across all cartilage zones in the vehicle group, consistent with severe matrix depletion. In the implant group, staining intensity was preserved, particularly within the middle and deep cartilage zones, indicating attenuation of extracellular matrix degradation (Figure 4A–C). Quantitative image analysis of the toluidine blue-positive area (Figure 4D) corroborated these findings. The stained area was significantly reduced in the vehicle group compared with the sham group (*p* = 2.05 × 10^−5^). Notably, the implant group exhibited a significantly greater toluidine blue-positive area than the vehicle group (*p* = 0.023). Although matrix preservation in the implant group did not fully return to sham levels, DFAT treatment resulted in an approximately 1.7-fold increase in proteoglycan retention relative to untreated OA joints.

### 3.4. Elevation of Prostaglandin E_2_ in Synovial Fluid

To provide in vivo molecular evidence for paracrine activity within the joint space, we quantified prostaglandin E_2_ (PGE_2_) concentrations in synovial fluid at Day 84. As shown in Figure 5, DFAT cell transplantation led to a significant increase in synovial fluid PGE_2_ levels compared to both the sham and vehicle groups. PGE_2_ is a well-established immunosuppressive eicosanoid, and previous studies have demonstrated that its production and secretion by MSCs contribute to the resolution of inflammation across various chronic inflammatory diseases, including arthritis [28], tissue injury [29], autoimmune uveoretinitis [30], and acute liver failure [31]. Taken together, these findings indicate that DFAT administration promotes the intra-articular release of immunosuppressive eicosanoids to suppress inflammatory responses.

## 4. Discussion

The present study demonstrates that i.a. administration of allogeneic DFAT cells exerts significant chondroprotective effects in a canine MIA-induced OA model. Compared with rodent models, canine models offer substantially greater translational relevance due to their anatomical, biomechanical, and pathological similarities to humans, including comparable joint size, cartilage thickness, and a natural propensity to develop spontaneous OA [1]. The successful attenuation of cartilage erosion in a weight-bearing large-animal joint therefore provides strong preclinical evidence supporting the clinical applicability of DFAT-based regenerative therapy [2].

The most pronounced macroscopic improvements were observed in the femoral trochlea. In canine knees, the trochlea is exposed to considerable shear stress during locomotion [32]. In cases of naturally occurring OA associated with cranial cruciate ligament (CCL) disease, cartilage lesions in the trochlea have been reported in 34 of 40 affected stifles [33]. Similarly, experimental transection of the CCL results in cartilage hypertrophy in the trochlear region [34,35]. The ability of DFAT cells to preserve cartilage integrity in this mechanically vulnerable region suggests that the therapy may not only provide biological support but also enhance structural resilience under repetitive mechanical loading. This region-specific protection highlights the therapeutic robustness of DFAT transplantation in functionally demanding joint compartments.

Histopathological findings further support the therapeutic effect of DFAT cells. The preservation of toluidine blue metachromasia in the middle and deep cartilage zones indicates maintenance of GAG content and ECM integrity. Consistent with previous studies on mesenchymal progenitor cells [36], the stabilization of ECM components likely reflects paracrine mechanisms rather than direct structural integration of transplanted cells [5]. In rodent models, DFAT cells express cartilage-protective genes such as PRG4 and BMP6. Under inflammatory stimulation (e.g., tumor necrosis factor-α or interferon-γ exposure), DFAT cells upregulate genes associated with immune modulation, anti-inflammatory activity, and cartilage protection, including PTGS2, TNFAIP6, and BMP2 [5]. Our synovial fluid analysis strongly supports this paracrine mechanism; specifically, intra-articular PGE_2_ levels—a functional mediator downstream of PTGS2—were significantly elevated in the implant group compared with both the sham and vehicle groups. Given that PGE_2_ secreted by implanted cells plays a crucial role in the suppression of inflammatory cascades and promoting endogenous tissue repair, these in vivo molecular findings demonstrate that implanted DFAT modulates the intra-articular biochemical microenvironment to protect deep zone chondrocytes and the osteochondral interface from MIA-induced damage via the release of immune-suppressive lipid mediators.

The safety profile observed in this study further strengthens the therapeutic potential of allogeneic DFAT transplantation. The absence of sustained joint swelling, functional deterioration, or systemic abnormalities following injection suggests that allogeneic DFAT cells do not provoke intra-articular immune reactions [37,38]. This immunological tolerance is particularly advantageous for clinical translation, as it enables an “off-the-shelf” therapeutic strategy. Such an approach allows immediate availability of standardized, quality-controlled DFAT cell products while avoiding the limitations associated with autologous fat harvesting and individualized cell processing.

Despite these encouraging findings, several limitations of this study warrant consideration. First, the sample size was relatively small, which may limit the generalizability of the findings. However, a post hoc power analysis based on the observed effect size for the primary outcome (total OARSI score; Cohen’s *f* = 0.988) revealed a statistical power (1 − β) of 98.6% at a significance level α = 0.05, indicating that the therapeutic effect of DFAT transplantation was sufficiently robust to overcome individual variation in this model. Nevertheless, these results should be interpreted as preliminary proof-of-concept evidence, and future studies with larger sample sizes and longer follow-up periods are warranted to assess the durability of cartilage preservation and therapeutic efficacy. Second, while histological evaluations adhered strictly to mandatory OARSI consensus guidelines using H&E and Toluidine Blue staining, specific matrix protein profiling (such as immunohistochemistry for Collagen Type II and Aggrecan) was not performed in this study. Third, the extent of chondrogenic differentiation potential and cell fate of the transplanted DFAT remains to be fully defined. In vivo tracing experiments and complete characterization of chondrogenic potency in vitro and in vivo are underway in our laboratory. To deepen mechanistic insights, clarify chondrogenic reprogramming, and optimize the therapeutic efficacy of canine DFAT cells for clinical translation, comprehensive investigations are currently ongoing in our laboratory.

## 5. Conclusions

This study provides translationally relevant evidence that i.a. allogeneic DFAT cell therapy preserves cartilage structure and extracellular matrix integrity in a clinically meaningful large-animal OA model. These findings support continued development of DFAT-based regenerative approaches for both veterinary and human osteoarthritis.

## Figures and Tables

**Table 1:** A comprehensive hematological analysis.

Group	WBC (10^2^/μL)	RBC (10^4^/μL)	HGB (g/dL)	HCT (%)	MCV (fL)	MCH (pg)	MCHC (g/dL)	PLT (10^4^/μL)	LY (%)	MO (%)	EO (%)	GR (%)	RDWCV (%)	RDWSD (fL)	PCT (%)	MPV (fL)	PDW (%)
sham	93	623	14.2	38.8	62.3	22.8	36.6	52.9	14.6	4	0.3	81.1	13.4	33.4	0.35	6.6	15.4
sham	102	636	15.5	41.4	65.1	24.4	37.4	41.5	20.5	4	0.6	74.9	13.3	34.6	0.32	7.6	15.3
sham	79	649	15.7	41.9	64.6	24.2	37.5	42.6	25.9	4	0.8	69.3	11.9	30.7	0.29	6.9	15.8
vehicle	109	709	16.3	44.7	63	23	36.5	43.5	20.4	3.8	1.1	74.7	13	32.8	0.31	7.2	15.1
vehicle	95	843	19.9	53.6	63.6	23.6	37.1	38.3	25.4	2.9	0.9	70.8	12.7	32.3	0.28	7.2	15.6
vehicle	82	657	15.6	42.2	64.2	23.7	37	53.6	23.2	4.2	0.9	71.7	12.7	32.6	0.37	6.9	15.8
vehicle	108	741	17.5	47	63.4	23.6	37.2	37.8	18	3.1	1.6	77.3	13.3	33.7	0.29	7.7	15
vehicle	98	822	19.4	50.9	61.9	23.6	38.1	38	38.8	3.3	1.1	56.8	12.8	31.7	0.3	7.9	14.7
vehicle	80	661	16.3	42.8	64.8	24.7	38.1	30.4	39.2	4.5	1.1	55.2	12.3	31.9	0.22	7.4	14.8
implant	90	744	18.2	48.2	64.8	24.5	37.8	40.4	18.2	2.8	1.1	77.9	13.2	34.2	0.33	8.1	15.3
implant	94	730	17.2	45.6	62.5	23.6	37.7	47.2	15	3.8	1.1	80.1	13.9	34.8	0.35	7.4	15.7
implant	64	751	16.6	45.1	60.1	22.1	36.8	57.9	23.1	4.6	2.3	70	12.8	30.8	0.45	7.7	15

EO: sham-implant: 0.043.

**Table 2:** Systemic histopathology.

Group	Heart	Lung	Liver	Spleen	Kidney
sham	NP	NAD	NAD	NAD	NAD
	NP	NAD	NAD	NAD	NAD
	NP	NAD	Lymphocytic plasmacytic cholangitis (mild)	NAD	NAD
vehicle	NAD	Suppurative bronchopneumonia (mild)	Suppurative cholangitis (mild)	NAD	NAD
	NAD	NAD	Suppurative hepatitis (mild)	NAD	NAD
	NAD	NAD	NAD	NAD	NAD
	NAD	NAD	NAD	NAD	NAD
	NAD	NAD	NAD	NAD	NAD
	NAD	NAD	NAD	NAD	NAD
implant	NAD	NAD	NAD	NAD	medullary nephrocalcinosis (mild)
	NAD	NAD	NAD	NAD	NAD
	NAD	NAD	NAD	NAD	NAD

NP, not performed. NAD, nothing abnormal detected.

## Data Availability

The original contributions presented in this study are included in the article. Further inquiries can be directed to the corresponding author.
